# Multi-modal domain adaptation for revealing spatial functional landscape from spatially resolved transcriptomics

**DOI:** 10.1093/bib/bbae257

**Published:** 2024-05-31

**Authors:** Lequn Wang, Yaofeng Hu, Kai Xiao, Chuanchao Zhang, Qianqian Shi, Luonan Chen

**Affiliations:** Key Laboratory of Systems Biology, Shanghai Institute of Biochemistry and Cell Biology, Center for Excellence in Molecular Cell Science, Chinese Academy of Sciences, No. 320 Yue Yang Road, Xuhui District, Shanghai 200031, China; University of Chinese Academy of Sciences, No. 80 Zhongguancun East Road, Haidian District, Beijing 100049, China; Key Laboratory of Systems Health Science of Zhejiang Province, School of Life Science, Hangzhou Institute for Advanced Study, University of Chinese Academy of Sciences, 1 Xiangshan Lane, Hangzhou 310024, China; Key Laboratory of Systems Biology, Shanghai Institute of Biochemistry and Cell Biology, Center for Excellence in Molecular Cell Science, Chinese Academy of Sciences, No. 320 Yue Yang Road, Xuhui District, Shanghai 200031, China; University of Chinese Academy of Sciences, No. 80 Zhongguancun East Road, Haidian District, Beijing 100049, China; Key Laboratory of Systems Health Science of Zhejiang Province, School of Life Science, Hangzhou Institute for Advanced Study, University of Chinese Academy of Sciences, 1 Xiangshan Lane, Hangzhou 310024, China; Hubei Engineering Technology Research Center of Agricultural Big Data, Huazhong Agricultural University, No. 1 Shizishan Street, Hongshan District, Wuhan 430070, Hubei Province, China; Hubei Key Laboratory of Agricultural Bioinformatics, College of Informatics, Huazhong Agricultural University, No. 1 Shizishan Street, Hongshan District, Wuhan 430070, Hubei Province, China; Key Laboratory of Systems Biology, Shanghai Institute of Biochemistry and Cell Biology, Center for Excellence in Molecular Cell Science, Chinese Academy of Sciences, No. 320 Yue Yang Road, Xuhui District, Shanghai 200031, China; University of Chinese Academy of Sciences, No. 80 Zhongguancun East Road, Haidian District, Beijing 100049, China; Key Laboratory of Systems Health Science of Zhejiang Province, School of Life Science, Hangzhou Institute for Advanced Study, University of Chinese Academy of Sciences, 1 Xiangshan Lane, Hangzhou 310024, China

**Keywords:** spatially resolved transcriptomics, spatial domain identification, unsupervised domain adaptation, spatial distribution alignment

## Abstract

Spatially resolved transcriptomics (SRT) has emerged as a powerful tool for investigating gene expression in spatial contexts, providing insights into the molecular mechanisms underlying organ development and disease pathology. However, the expression sparsity poses a computational challenge to integrate other modalities (e.g. histological images and spatial locations) that are simultaneously captured in SRT datasets for spatial clustering and variation analyses. In this study, to meet such a challenge, we propose multi-modal domain adaption for spatial transcriptomics (stMDA), a novel multi-modal unsupervised domain adaptation method, which integrates gene expression and other modalities to reveal the spatial functional landscape. Specifically, stMDA first learns the modality-specific representations from spatial multi-modal data using multiple neural network architectures and then aligns the spatial distributions across modal representations to integrate these multi-modal representations, thus facilitating the integration of global and spatially local information and improving the consistency of clustering assignments. Our results demonstrate that stMDA outperforms existing methods in identifying spatial domains across diverse platforms and species. Furthermore, stMDA excels in identifying spatially variable genes with high prognostic potential in cancer tissues. In conclusion, stMDA as a new tool of multi-modal data integration provides a powerful and flexible framework for analyzing SRT datasets, thereby advancing our understanding of intricate biological systems.

## Introduction

Recent advancements in spatially resolved transcriptomics (SRT) technologies, such as MERFISH [1], STARmap [2], 10x Visium and Slide-seqV2 [3], have enabled the capturing of mRNA within spatial contexts. These diverse SRT technologies contribute to uncover the complex transcriptional landscape and functional regions in tissue, which are crucial for the study of complex disease [4–6]. However, due to the technical limitations, the expression measurements in SRT data are often sparse and noisy, posing significant challenges in deciphering the spatial functional regions. To address this tissue, additional modalities (e.g. histological images and spatial locations) are utilized to mitigate potential noise or bias in expression measurements. Nonetheless, accurately modeling the available SRT modalities for spatial domain detection still remains challenging due to sparse and noisy data problem.

Spatial domain detection plays a fundamental role in SRT data analysis. It entails the identification of clusters of spots exhibiting similar gene expression patterns and spatial coherence. These spatial domains often correspond to regions with similar biological functions, thereby playing a crucial role in downstream analyses such as identifying spatially variable genes (SVGs) and discovering prognostic genes in tumor tissues. To effectively tackle spatial domain detection, several computational methods have been developed. For example, STAGATE utilizes an autoencoder with an attention mechanism to adaptively learn the similarity of neighboring spots, which can refine the boundaries of different spatial domains [7]. SpaGCN employs a graph convolutional network (GCN) to promote the inclusion of nearby spots in the same cluster [8]. DeepST employs a variational graph autoencoder to extract the final latent embeddings for identifying spatial domains [9]. Although SpaGCN and DeepST incorporate information from histological images, neither approach models the histological images as a separate modality for learning effective representations. Specifically, SpaGCN treats histological images as an additional spatial coordinate axis or *z*-axis, whereas DeepST solely utilizes these images to augment the gene expression matrix during preprocessing. It is worth noting that these existing methods primarily rely on single models, which may have limitations in effectively handling complex datasets across various platforms and species. In contrast, utilizing multi-model approaches offers the potential for increased robustness and improved performance in such scenarios.

In this study, to solve the sparse and noisy problem of multi-modal data, we propose stMDA, a novel multi-modal unsupervised domain adaptation approach designed to integrate gene expression, histological images and spatial locations for revealing spatial functional regions. Specifically, stMDA first utilizes multiple neural network architectures to learn modality-specific representations from spatial multi-modal data and next aligns the global and spatially local distributions of spots across these modality-specific representations. Then, stMDA integrates these multi-modal representations to reconstruct gene expression by employing an attention mechanism and a decoder component. In particular, the deep spatial distributed alignment offered by stMDA represents a weak coupling approach that integrates multi-modal data based on clustering assignments, thereby enhancing the consistency of clustering results. In this manner, stMDA not only corrects low-quality gene expression from other modalities but also maximizes the benefits of multiple neural network architectures in processing diverse modalities to enhance the performance of spatial domain detection.

Our comprehensive evaluation showcases the superior and robust performance of stMDA on a diverse set of 17 datasets obtained from various SRT platforms. These datasets consist of 14 profiles obtained through the 10x Visium platform, 2 from the Slide-seqV2 platform and 1 from the Stereo-seq platform. The excellence of stMDA becomes evident when identifying spatial domains in the challenging 12 dorsolateral prefrontal cortex (DLPFC) datasets with known ground truth annotations. In this context, stMDA consistently outperforms other methods in terms of accuracy. In the context of tumor analysis, stMDA effectively identifies tumor regions on a pancreatic ductal adenocarcinoma (PDAC) tissue slice, with support from marker genes. Moreover, it excels at identifying spatially variable genes (SVGs) that hold prognostic significance, offering valuable insights into the clinical implications of gene expression patterns. The versatility of stMDA is evident in its ability to analyze well-structured tissue slices across various resolutions and species, including human and mouse embryos, the mouse olfactory bulb and the mouse hippocampus. Collectively, these results establish stMDA as a new potent and versatile tool for integrating multi-modal SRT data.

## Methods

### Overview of stMDA

**Figure 1:**
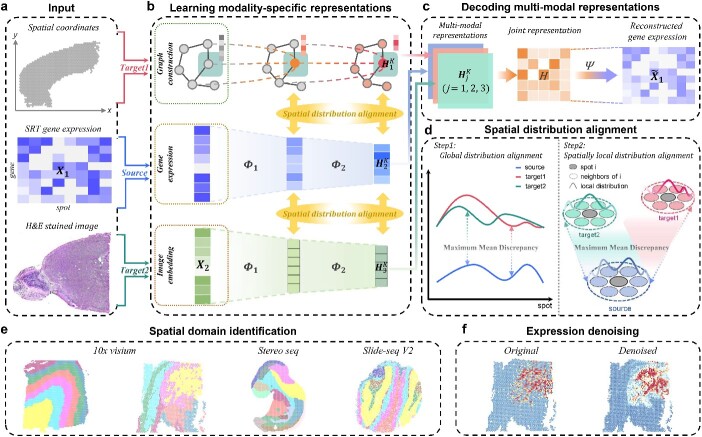
Schematic overview of stMDA. This figure illustrates the structure of stMDA, a multi-modal unsupervised domain adaptation with deep spatial distribution alignment. (**A**) The three data sources for stMDA inputs are SRT expression matrix, spatial coordinates and hematoxylin and eosin (H&E) staining image. stMDA considers gene expression (i.e. ${X}_1$) and other modal data (i.e. histological images and spatial locations) as the source and target domain datasets, respectively. (**B**) stMDA first learns modality-specific representations from spatial multi-modal data using a GCN, an encoder component and a CNN. To integrate multi-modal data based on clustering assignments, stMDA employs an innovative deep spatial distribution alignment strategy during representation learning. (**C**) Then, stMDA uses attention mechanism to obtain the joint representation (i.e. $H$) from these modality-specific representations (i.e. ${H}_1^{(K)},{H}_2^{(K)}$, ${H}_3^{(K)}$) and further reconstruct gene expression (i.e. ${\tilde{X}}_1$) using a decoder component. (**D**) The spatial distribution alignment improves the consistency of the spatially local and global distribution of each spot in the modality-specific representations using MMD. (**E**, **F**) Biological applications for stMDA include spatial domain identification and data denoising. (**E**) The joint representation (i.e. $H$) can be applied to detect spatial domains. (**F**) The reconstructed expression ${\tilde{X}}_1$ can be employed to denoise data.

Considering the data characteristics of different spatial transcriptomics (SRT) modalities, stMDA constructs multiple neural network architectures with deep spatial distribution alignment, integrates the multiple modalities and learns an effective joint representation. This capability allows stMDA to denoise expression measurements and identify spatial domains as a tool of multi-modal data integration (Fig. 1).

Specifically, stMDA considers gene expression (i.e. ${X}_1\in{R}^{M\times N}$, $M$ and $N$ are, respectively, the number of gene and spot) and other modalities (i.e. histological images and spatial locations) as the source and target domain datasets, respectively (Fig. 1A). Next, stMDA learns modality-specific representations from spatial multi-modal data using a GCN, an encoder component (i.e. $q\left({H}_2^{(K)}|{X}_1\right)$ where ${H}_2^{(K)}$ is the latent representation from gene expression) and a convolutional neural network (CNN) (Fig. 1B). To integrate multi-modal data based on clustering assignments, stMDA employs an innovative deep spatial distribution alignment strategy (Fig. 1B and D). This strategy improves the consistency of the spatially local and global distribution of each spot in the modality-specific representations. Finally, stMDA decodes these modality-specific representations (i.e. ${H}_1^{(K)},{H}_2^{(K)}$and ${H}_3^{(K)}$ where $K$ is the number of encoder layers) to obtain the joint representation (i.e. $H$) and reconstruct gene expression (i.e. ${\tilde{X}}_1$) using the attention mechanism and a decoder component (i.e. $p\left({X}_1|H\right)$) (Fig. 1C). In summary, stMDA learns effective representations from the source domain dataset and integrates the information from the target domain datasets.

After the training is complete, the converged joint latent representation (i.e. $H$) and the reconstructed gene expression matrix (i.e. ${\tilde{X}}_1$) are utilized for downstream analysis. The joint latent representation $H$ is utilized to construct a nearest neighbor network, which is then integrated with the Leiden [10] algorithm to identify spatial domains. Simultaneously, the reconstructed expression matrix ${\tilde{X}}_1$ functions as a denoised expression profile, enhancing spatial expression patterns and domain specificity. This denoised data are further employed to identify genes of biological interest.

### Building multi-modal unsupervised domain adaptation

#### Learning the spatial coordinates-specific representation

We convert spatial locations into a weighted graph $A$, which denotes the relationship between each spot and its spatial neighbors. To use the valuable spatially local structure, we introduce the GCN [11] to focus on the graph $A$.

- *Constructing graph-structure data*: We first select the *k*-nearest spatial neighbors of each spot by calculating the Euclidean distance of each pair of spots based on the spatial coordinates. Then, we calculate the similarity matrix of these neighboring spots based on PCA embedding (i.e. PCs) of expression (i.e. ${X}_1\in{R}^{M\times N}$).

(1) \begin{equation*} {A}_{ij}=\frac{D_{ij}}{\sum_{i=0}^N{D}_{ij}},{D}_{i,j}=\mathit{\exp}\left(2-\frac{\left\langle{U}_i,{U}_j\right\rangle }{\left\Vert{U}_i\right\Vert \bullet \left\Vert{U}_j\right\Vert}\right) \end{equation*}

where matrix $U\in{R}^{15\times N}$ is a low-dimensional matrix consisting of 15 PCs. ${U}_i$ is the top 15 principal components of spot $i$. $\left\langle{U}_i,{U}_j\right\rangle$ denotes the Euclidean distance between ${U}_i$ and ${U}_j$. $\left\Vert{U}_i\right\Vert$ represents the module of vector ${U}_i$. Matrix $D$ is the cosine distance of these neighboring spots.

- *Learning representation via GCN*: The representation learned by the $l$-th layer of the GCN from the graph-structure data (i.e. target domain dataset 1: $G\left({X}_1,A\right)$), denoted as ${H}_1^{(l)}$, is calculated as follows:

(2) \begin{equation*} {\displaystyle \begin{array}{c}{H}_1^{(l)}={\phi}_l\left({W}^{(l)}{H}_1^{\left(l-1\right)}{\tilde{D}}^{-\frac{1}{2}}{\tilde{A}}{\tilde{D}}^{-\frac{1}{2}}+{b}^{(l)}\right),l=1,\dots, K,\end{array}} \end{equation*}

where ${\phi}_l$ represents the activation function of the $l$-th layer within the GCN. ${W}^{(l)}$ denotes the weight matrix. ${b}^{(l)}$ corresponds to the bias term. ${\tilde{A}}$ is the normalized adjacency matrix, defined as ${\tilde{A}}=A+I$, with $I$ being the identity diagonal matrix. ${\tilde{D}}_{ii}$ is calculated as ${\sum}_j{\tilde{A}}_{ij}$. The term ${\tilde{D}}^{-\frac{1}{2}}{\tilde{A}}{\tilde{D}}^{-\frac{1}{2}}$ represents the normalized adjacency matrix. $K$ is the number of layers in GCN. For convenience, we denote the gene expression matrix ${X}_1$ as ${H}_1^{(0)}$.

#### Learning the gene expression–specific representation

As the source domain dataset, we employ the encoder network of variational autoencoder (VAE) to extract representations exclusively from the gene expression data (i.e. ${X}_1$). We calculate the specific representation ${H}_2^{(l)}$ of gene expression in the $l$-th layer encoder as follows:


(3) \begin{equation*} {\displaystyle \begin{array}{c}{H}_2^{(l)}={\phi}_l\left({W}^{(l)}{H}_2^{\left(l-1\right)}+{b}^{(l)}\right),\kern0.75em l=1,\dots, K\end{array}} \end{equation*}

where we denote the gene expression matrix ${X}_1$ as ${H}_2^{(0)}$.

#### Learning the histological image–specific representation

We first segment the histology image (i.e. target domain dataset 2) according to the spatial coordinates of each spot to obtain its partial image. Then, we transform each spot image into 2048-dimensional latent variables (i.e. ${X}_2\in{R}^{2048\times N}$) by using a pretrained CNN (i.e. ResNet-50 [12]). To learn the specific representation of this modality, the $l$-th layer representation, denoted as ${H}_3^{(l)}$, is computed as follows:


(4) \begin{equation*} {\displaystyle \begin{array}{c}{H}_3^{(l)}={\phi}_l\left({W}^{(l)}{H}_3^{\left(l-1\right)}+{b}^{(l)}\right),l=1,\dots, K,\end{array}} \end{equation*}

where we denote the gene expression matrix ${X}_2$ as ${H}_3^{(0)}$.

#### Deep spatial distribution alignment

To effectively integrate the spatial multi-modal data, we align the distributions of the modality-specific representations within spatial context by providing deep spatial distribution alignment strategy.

- *Aligning the global distribution of the modality-specific representation*: The distribution of spots can measure clustering assignments. We align the distribution of the modality-specific representations to ensure that stMDA learns the consistent clustering results from spatial multi-modal data. We quantify the distribution difference between source domain dataset (i.e. gene expression) and two target domain datasets (i.e. spatial locations information and histological images) via Maximum Mean Discrepancy (MMD) [13].

(5) \begin{align*}{Loss}_{alig{n}_D}=\sum_{l=1}^K MMD\left({H}_2^{(l)},{H}_1^{(l)}\right)+ MMD\left({H}_2^{(l)},{H}_3^{(l)}\right) \end{align*}

(6) \begin{align*}MMD\left(X,Y\right)=&\ {\left\Vert \frac{1}{n}\sum_{i=1}^n\phi \left({x}_i\right)-\frac{1}{n}\sum_{j=1}^n\phi \left({y}_j\right)\right\Vert}_H^2\nonumber\\=&\ \frac{1}{n^2}\sum_{i=1}^n\sum_{j=1}^nK\left({x}_i,{x}_j\right)-\frac{1}{n^2}\sum_{i=1}^n\sum_{j=1}^nK\left({x}_i,{y}_j\right)\nonumber\\&+\frac{1}{n^2}\sum_{i=1}^n\sum_{j=1}^nK\left({y}_i,{y}_j\right) \end{align*}

where $K\left(x,y\right)={e}^{-\frac{{\left\Vert x-y\right\Vert}^2}{2}}$ is the Gaussian kernel function. The $\phi \left(\bullet \right)$ function maps the data into a regenerative Hilbert space. ${\left\Vert \bullet \right\Vert}_H^2$ indicates that this distance is measured by data in the regenerated Hilbert space.

- *Aligning the spatially local distribution of each spot*: Considering the influence of spatial context on spot clustering, spatial neighbors of each spot should be the consistent clustering assignments between source-specific representation and target-specific representations. We use MMD to quantify the spatially local distribution difference of each spot between source and target domain datasets.

(7) \begin{align*}{Loss}_{alig{n}_{SD}}=& {}\sum_{l=1}^K\sum_{i=1}^N MMD\left({\left({H}_2^{(l)}\right)}_i^{Neighbor},{\left({H}_1^{(l)}\right)}_i^{Neighbor}\right)\nonumber\\&+ MMD\left({\left({H}_2^{(l)}\right)}_i^{Neighbor},{\left({H}_3^{(l)}\right)}_i^{Neighbor}\right) \end{align*}

where ${\left({H}_1^{(l)}\right)}_i^{Neighbor},{\left({H}_2^{(l)}\right)}_i^{Neighbor},{\left({H}_3^{(l)}\right)}_i^{Neighbor}$ is composed of the spatial neighbors of the *i*-th spot in $l$-th layer representation of spatial locations, gene expression and histological images, respectively.

#### Decoding the multi-modal representations

We decodes the learned multi-modal representations to obtain the effective joint representation. The main procedure can be stated as follows.

- *Integrating the modality-specific representations*: To consolidate the information learned from other modalities, we perform the attention mechanism [14] to integrate the modality-specific representations (i.e. ${H}_1^{(K)},{H}_2^{(K)}$ and ${H}_3^{(K)}$) and get the joint representation (i.e. $H$). Here, we focus on spot $i$ (i.e. ${\left({H}_1^{(K)}\right)}_i\in{R}^{D\times 1},{\left({H}_2^{(K)}\right)}_i\in{R}^{D\times 1}\ and\ {\left({H}_3^{(K)}\right)}_i\in{R}^{D\times 1}$) and use one shared attention vector ${W}_{att}\in{R}^{D^{\prime}\times 1}$ to get the attention values $\left[{\alpha}_1^i,{\alpha}_2^i,{\alpha}_3^i\ \right]$ as follows:

(8) \begin{align*}\left[{\alpha}_1^i,{\alpha}_2^i,{\alpha}_3^i\ \right]=&\ softmax\left({W_{att}}^T\bullet \mathit{\tanh}\left(W\left[{\left({H}_1^{(K)}\right)}_i,{\left({H}_2^{(K)}\right)}_i,{\left({H}_3^{(K)}\right)}_i\right]\right.\right.\nonumber\\&+\left[b,b,b\right]\Big)\Big) \end{align*}

where $W\in{R}^{D^{\prime}\times D}$ is the weight matrix and $b\in{R}^{D^{\prime}\times 1}$ is the bias vector. For all spots, we have the learned attention weight ${\alpha}_1=\mathit{\operatorname{diag}}\left(\left({\alpha}_1^1,\cdots{\alpha}_1^i,\cdots{\alpha}_1^N\right)\right)$, ${\alpha}_2=\mathit{\operatorname{diag}}\left(\left({\alpha}_2^1,\cdots{\alpha}_2^i,\cdots{\alpha}_2^N\right)\right)$ and ${\alpha}_3=\mathit{\operatorname{diag}}\left(\left({\alpha}_3^1,\cdots{\alpha}_3^i,\cdots{\alpha}_3^N\right)\right)$. Then, we obtain joint representation by combing the modality-specific representations as follows:


(9) \begin{equation*} {\displaystyle \begin{array}{c}H={H}_1^{(K)}{\alpha}_1+{H}_2^{(K)}{\alpha}_2+{H}_3^{(K)}{\alpha}_3\end{array}} \end{equation*}

- *Decoding the joint representation*: Following the encoding process, the joint representation (i.e. $H$) is decoded using a dedicated decoder network to reconstruct the gene expression matrix. In this setup, assuming a total of $L$ layers, the decoder network encompasses layers from $K+1$ to $L$. The $l$-th layer representation, denoted as ${H}^{(l)}$, is computed as follows:

(10) \begin{equation*} {\displaystyle \begin{array}{c}{H}^{(l)}={\psi}_l\left({W}^{(l)}{H}^{\left(l-1\right)}+{b}^{(l)}\right),\kern0.5em l=K+1,\dots, L\end{array}} \end{equation*}

where ${\psi}_l$ represents the activation function of the $l$-th layer within the decoder. ${W}^{(l)}$ denotes the weight matrix. ${b}^{(l)}$ corresponds to the bias term. We denote the gene expression matrix $H$ as ${H}^{(K.)}$

Combined with the loss (i.e. ${\mathcal{L}}_{ELBO}$) of VAE, the overall loss function of stMDA is denoted as follows:


(11) \begin{equation*} \!\!\!{\displaystyle \begin{array}{c}{\mathcal{L}}_{ELBO}=-{E}_{q\left({H}_2^{(K)}|{X}_1\right)}\left( logp\left({X}_1|H\right)\right)+ KL\left(q\left({H}_2^{(K)}|{X}_1\right)\left\Vert p\left({H}_2^{(K)}\right)\right.\right)\end{array}} \end{equation*}

(12) \begin{equation*} {\displaystyle \begin{array}{c}\mathcal{L}={\mathcal{L}}_{ELBO}+\lambda \left({Loss}_{alig{n}_D}+{Loss}_{alig{n}_{SD}}\right)\end{array}}\, \qquad\qquad\qquad\qquad\end{equation*}

We have provided the parametric sensitivity analysis of λ using DLPFC to discuss its impact on domain detection (Supplementary Figure 1), and the tunable parameter $\lambda$ can be manually set and defaults to 1.

### Hyperparameter tunings and implementations

In the stMDA model, we maintained a consistent network structure across all datasets, specifically using 128 neurons in the hidden layer and 10 neurons in the latent layer. We employed the Adam optimizer with its default settings in PyTorch for optimization.

We recommend to tune the parameter λ according to different contexts. A fixed value of λ is not universally applicable across all datasets due to varying sensitivities to the changes in λ, which controls the degree of spatial distribution alignment or the utilization of spatial information. A small value of λ results in less spatial distribution alignment, and setting λ to 0 completely disables spatial distribution alignment, and histological images information cannot affect representation learning. We initiate the search for λ from a small value, determining the optimal range of λ ∈ [0,3] through grid search and cross-validation. The primary goal of stMDA is to identify spatial regions that align closely with pathological characteristics and physiological structure. With this motivation as a priori, we select λ by maximizing unsupervised cluster evaluation measurements such as the adjusted Rand index (ARI) or purity. In situations where datasets lack manual annotations but include reference diagrams, we provide a search range for parameter λ tailored to the SRT data from different platforms. Based on these ranges, the optimal λ parameter is determined by the difference between output results and reference diagrams (Supplementary Table 1).

### Data preprocessing

For 10x Visium, Slide-seqV2 and Stereo-seq datasets, the top 3000 highly variable genes (HVGs) were selected using the scanpy.pp.highly_variable_genes() function from the SCANPY Python package. Additionally, log transformation was performed on the expression profiles using scanpy.pp.log1p() on the gene expression data.

### Spatial domain detection and gene expression denoising

stMDA leverages the joint latent representation $H$ to create a *k*-nearest neighbor network, which is achieved by constructing a neighborhood graph using scanpy.pp.neighbors(). Subsequently, stMDA detects spatial domains using the Leiden algorithm, implemented through scanpy.tl.leiden(). The ‘resolution’ parameter is adjustable to align with the number of manual annotations. Additionally, stMDA utilizes the reconstructed expression data ${\tilde{X}}_1$ for gene expression denoising, enhancing the spatial expression profiles.

### Performance evaluation

For datasets that have manual spot annotations from previous studies, we evaluate the accuracy of the identified spatial domains using the ARI [15–20]. The ARI measures the agreement between the identified spatial domains and the manually annotated domains.

In cases where datasets lack manual annotations but include reference diagrams, we assess the agreement between the identified spatial domains and the reference diagrams. This evaluation helps determine the similarity and alignment between the identified domains and the expected spatial organization depicted in the reference diagrams. In addition to these evaluations, we utilize two internal metrics, i.e. silhouette coefficient (SC) [21] and Davies–Bouldin (DB) index [22], to assess the clustering performance based on spatial coordinates. The SC measures the spatial proximity of a spot to others within the same domain compared to those in different domains. We consider the mean value for all spots as the overall indicator, where a value close to 1 indicates appropriate clustering, while a value close to 0 indicates otherwise. On the other hand, the DB index quantifies the spatial proximity between domains, and in general, a lower value corresponds to better clustering.

To further support these assessments, we confirm the alignment of the identified spatial domains by examining the spatial expression patterns of marker genes reported in previous studies. This analysis allows us to validate that the identified domains correspond to the expected expression patterns of known marker genes, providing additional evidence for the accuracy and biological relevance of the identified spatial domains.

### Functional analysis of spatial domains

GSVA analysis [23] was performed on the identified spatial domains based on Hallmark gene set, and the differences of functional pathways in the identified spatial domains were analyzed. AUCell package performs activity scores of the interested biological hallmarks in each spot and maps the activity value of biological hallmarks to spatial coordinates for visually viewing the spatial functional landscape in tumor tissue.

### Survival analysis

To assess the prognostic significance of genes, we utilize bulk expression data with patient survival information. In the specific case of the PDAC cancer study, we perform Kaplan–Meier Plotter analysis [24], which allows us to evaluate the correlation between the expression levels of specific genes and patient survival outcomes and assess whether certain genes are associated with favorable or unfavorable prognosis.

## Results

### stMDA demonstrates superior accuracy in identifying spatial domains

We performed a comprehensive benchmarking analysis comparing stMDA to several competing methods using the datasets from the DLPFC. Maynard *et al.* meticulously annotated these DLPFC datasets based on cytoarchitecture and gene markers, assigning them to cortex layers 1–6 (L1–L6) and white matter (WM) [25]. During our benchmarking process, we used the annotation as a reference for ground truth, and the ARI served as the evaluation metric. The competing methods for spatial domain identification included BayesSpace [26], DeepST [9], GraphST [27], SEDR [28], SpaGCN [8], SpatialPCA [29] and STAGATE [7] (Supplementary Note S1).

**Figure 2:**
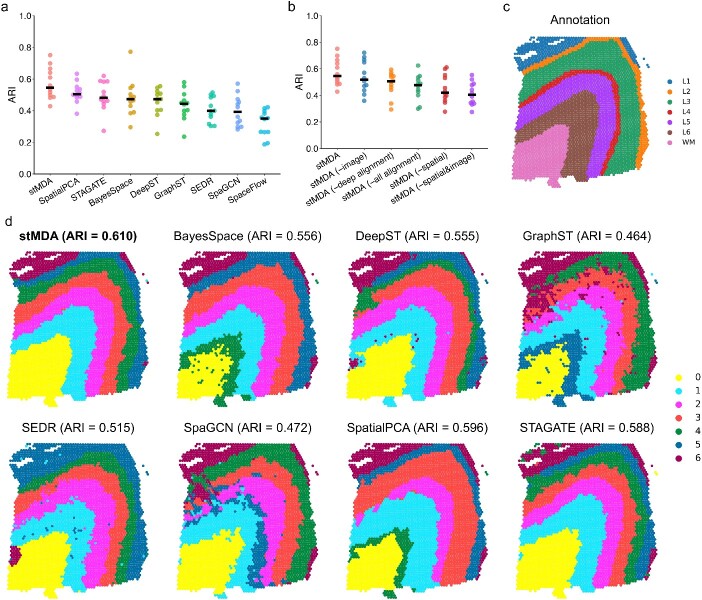
Performance comparison of spatial domain identification methods on 12 DLPFC datasets. (**A**) Displays the ARI values between the spatial domains identified by computational methods and the ground truth annotation. The bar highlights the mean ARI value for each method. (**B**) ARI boxplots of whether image information and spatial coordinates are used, whether deep spatial distribution alignment strategy is used in stMDA are shown. (**C**) Shows the manual annotation of slice 151 673 on the spatial coordinates. Annotations include L1–L6 (layer 1–layer 6) and WM. (**D**) Illustrates the spatial domains identified by stMDA and other competing methods on slice 151 673, displayed on the spatial coordinates. Each method’s spatial domain is color-coded for clarity, with the ARI value shown above.

The results clearly indicated the superiority of stMDA, as it achieved the highest mean ARI (mean ARI = 0.572) among the compared computational methods. Furthermore, this achievement was supported by a significant overall improvement in ARI compared to the alternative approaches (as confirmed by the Wilcoxon signed-rank test, *P* < 1e-4, as depicted in Fig. 2A and Supplementary Figure 2). To gain a more intuitive understanding of the spatial domains identified by stMDA and other methods, we examined slice 151 673. Importantly, this slice displayed a distinct layer structure consisting of cortex layers 1–6 (L1–L6) and WM, as depicted in Fig. 2C. In this case, the spatial domain identified by stMDA demonstrated the highest level of agreement with the ground truth annotation (ARI = 0.610). Moreover, it exhibited superior separation between layers, improved spatial continuity, minimal noise points and well-defined boundaries compared to the results obtained from other methods (as illustrated in Fig. 2D).

In this case, we also systematically evaluated the contribution of different components in the stMDA model to its overall performance (Fig. 2B). To assess the individual contributions of image information and spatial coordinates, we sequentially removed these components from the model and calculated the ARI values. We observed that ${\mathrm{stMDA}}^{-\mathrm{image}}$, which excluded image information, achieved higher ARI values compared to ${\mathrm{stMDA}}^{-\mathrm{spatial}}$, which excluded spatial coordinates, and ${\mathrm{stMDA}}^{-\mathrm{spatial}\&\mathrm{image}}$, which excluded both spatial coordinates and image information, obtained the lowest ARI values. However, the ARI values for these modified models were still lower than that of the complete stMDA model. This suggests that both images and spatial coordinates provide valuable information to the model, but spatial coordinates are particularly important. Spatial coordinates not only provide constraints of spatial local structure but also play a crucial role in the implementation of deep spatial distribution alignment strategy. Furthermore, we evaluated the performance of the deep spatial distribution alignment strategy by running stMDA without deep alignment (${\mathrm{stMDA}}^{-\mathrm{deep}\ \mathrm{alignment}}$) and without the entire deep spatial alignment (${\mathrm{stMDA}}^{-\mathrm{all}\ \mathrm{alignment}}$). We found that the deep spatial distribution alignment significantly improved the performance of stMDA.

In conclusion, our benchmarking analysis provides conclusive evidence of the exceptional accuracy and robust performance of stMDA in identifying spatial domains. This highlights its potential as a valuable tool for enhancing our comprehension of complex spatial transcriptomics data in comparison to existing methods.

### stMDA unveils the heterogeneity of pancreatic ductal adenocarcinoma tumors

**Figure 3:**
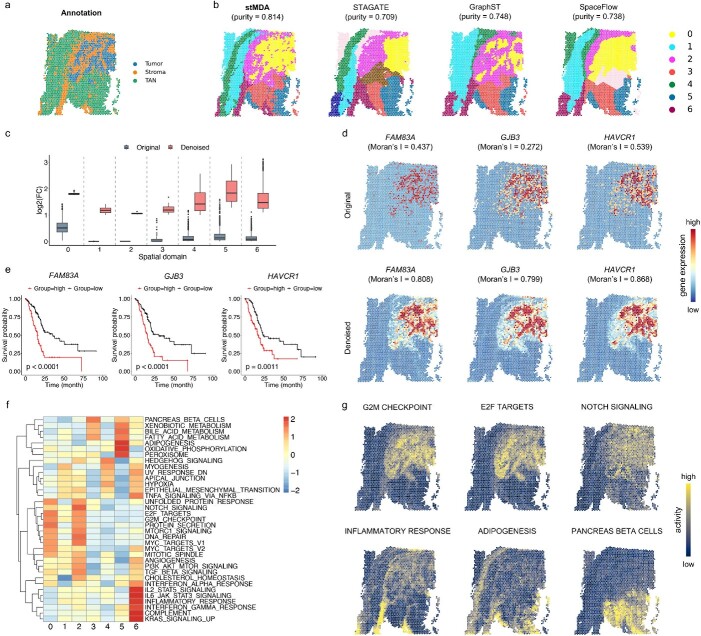
stMDA reveals tumor heterogeneity on the PDAC tumor slice. (**A**, **B**) Depicts the spatial domains identified by stMDA and other competing methods on the PDAC tumor slice, showcased on spatial coordinates. Each method’s spatial domain is color-coded for clarity, with rough correspondence. (**C**) Features a boxplot of log2 fold change values, measuring the differential expression pattern before and after denoising. In the boxplot, the center line represents the median, the box limits indicate the upper and lower quartiles and whiskers represent the 1.5× interquartile range. (**D**) Illustrates the original and denoised gene expression of spatially variable genes within the tumor region, displayed on spatial coordinates. The Moran’s I value of the gene expression before and after denoising is shown above. (**E**) Presents a Kaplan–Meier plot of genes as in (**D**), analyzed for survival on the PDAC clinical data. The *P*-value of the survival analysis is shown in the bottom-left corner. (**F**) Heat maps reflect the relevant biological hallmarks for each spatial domain. (**G**) The specific biological hallmarks of some spatial domains and the corresponding activity value in each spot.

Next, we aimed to showcase the robust performance of stMDA in identifying spatial domains and conducting denoising analysis within a PDAC tumor section [30]. This study aimed to demonstrate the adaptability of stMDA in analyzing highly heterogeneous tissue slices, thereby highlighting its effectiveness beyond well-structured datasets. The PDAC slice contained regions of tumor, normal tissue and tumor-adjacent normal (TAN) tissues (Fig. 3A). By utilizing stMDA for spatial domain identification, we compared its performance with other methods on this complex PDAC slice. Importantly, the spatial domains identified by stMDA exhibited enhanced separation, effectively distinguishing tumor, stroma and TAN tissues based on more accurate (stMDA, purity = 0.814; STAGATE, purity = 0.709; GraphST, purity = 0.748; SpaceFlow, purity = 0.738) and the corresponding marker genes (Fig. 3A and B and Supplementary Figures 3 and 4). For example, Domain 0 was predominantly categorized as tumor tissue, as indicated by the marker gene *GPX2* [31]. In contrast, Domain 2 and Domain 6 were associated with stroma tissues, exhibiting elevated expression of the marker gene *CFD* [32]. Domains 1, 3, 4 and 5 roughly corresponded to TAN tissues, characterized by *PGC*, *ACTG2* and *MUC13* [33] (Supplementary Figure 4). The spatial domain identification performed by stMDA displayed superior alignment with these marker genes compared to alternative methods, thereby underscoring its ability to identify biologically relevant spatial domains within heterogeneous tissues.

Subsequently, we utilized the reconstructed expression matrix for denoising to emphasize its impact on improving spatial expression patterns and domain expression specificity (Fig. 3C and D and Supplementary Figure 5). Domain expression specificity was quantified using log fold change, whereby a higher value indicated greater specificity to the respective domain. Spatial expression patterns were assessed using Moran’s I, which is a measure of spatial autocorrelation in gene expression. Higher values of Moran’s I indicate more coherent spatial patterns (Supplementary Figure 5). Remarkably, denoising significantly improved both spatial expression patterns and domain expression specificity across all domains (Fig. 3C, as confirmed by the Wilcoxon signed-rank test, *P* < 1e-15).

Moreover, the denoised expression data revealed underlying biological mechanisms, including the identification of genes with prognostic value and the functional analysis of the identified spatial domains. By applying a log fold change threshold of >1, we identified 409 differentially expressed genes (DEGs) within tumor domain 0 after denoising. In comparison, only 77 DEGs were identified before denoising, 42 of which demonstrated prognostic significance. For example, we highlighted *FAM83A* [34], *GJB3* [35] and *HAVCR1* [36] as illustrative examples to demonstrate the effectiveness of denoising and validate their prognostic value using independent the cancer genome atlas program (TCGA) clinical data (Fig. 3D and E). Remarkably, the spatial expression patterns became smoother after denoising, and quantitatively, the Moran’s I value for these genes increased substantially. Survival analysis on TCGA clinical data confirmed the association between high expression of these genes and a poor prognosis. To further reveal the tumor functional heterogeneity from the identified spatial domains, we quantified the activity value of each biological hallmark in each spatial domain and each spot and found the specific biological hallmarks of the identified spatial domains, further facilitating the revealing of functional heterogeneity in cancer tissue (Fig. 3F and G). For example, the domain 0 (tumor region) are enriched with G2M CHECKPOINT [37] and E2F TARGETS [38], which are associated with pancreatic cancer. The domain 2 (the adjacent stroma region of tumor region) are enriched with NOTCH SIGNALING, which is critical for cell proliferation, differentiation, development and homeostasis in pancreatic cancer [39]. INFLAMMATORY RESPONSE shows that domain 6 (stroma region) has a strong immune response to cancer development [40]. ADIPOGENESIS (in domain 5), PANCREAS BETA CELLS (in domain 3 and 5) tend to reflect the fact that domain 3, 5 are performing normal tissue functions [41–43]. Compared with the traditional rough division of cancer regions (Fig. 3A), the accurate analysis of spatial domains (Fig. 3B) can reveal the spatial functional landscape (Fig. 3D and F). Collectively, these results emphasize the robust performance of stMDA in handling heterogeneous data, striking a balance between biological relevance and spatial smoothness. The denoising analysis also offered new insights into the underlying biology of tumors.

### stMDA proficiently dissects human and mouse embryos across platforms

We present the exceptional versatility of stMDA in elucidating the intricate structures of human and mouse embryos across different platforms and species, highlighting its remarkable performance. To demonstrate stMDA’s robustness, we applied it to a mouse embryo slice profiled using Stereo-seq [44] and a human embryo slice profiled using 10x Visium technology [45].

**Figure 4:**
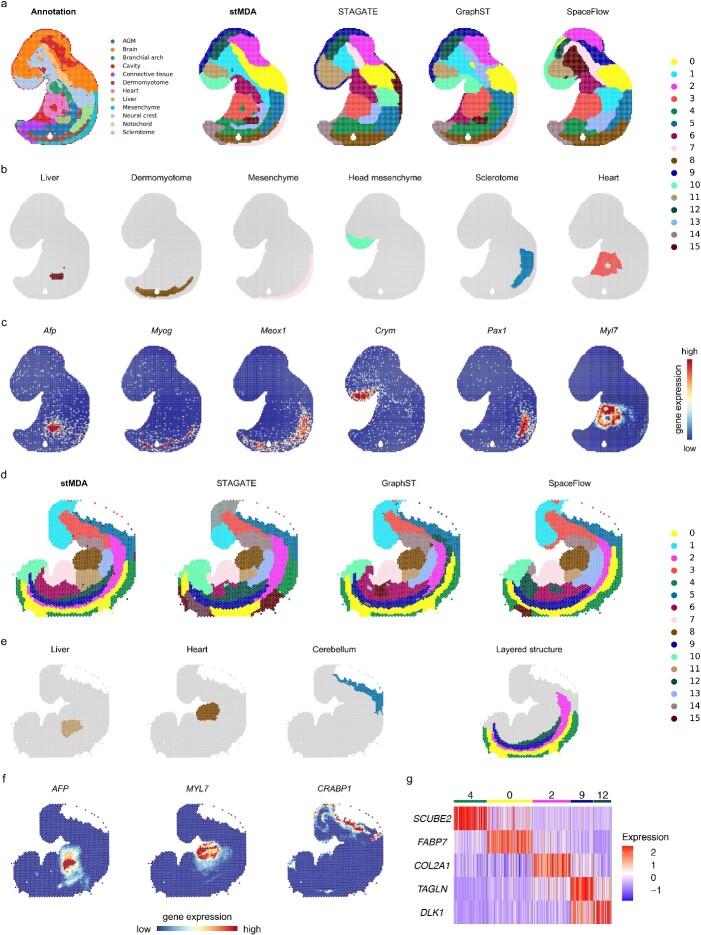
Spatial domain identification on mouse and human embryo datasets across platforms. (**A**) Depicts the manual annotation and the spatial domains identified by computational methods on the Stereo-seq mouse embryo dataset, showcased on spatial coordinates. Each method’s spatial domain is color-coded for clarity, with rough correspondence. (**B**) Offers a separate view of the focused spatial domains identified by stMDA, as shown in spatial coordinates. The spatial domains are colored as in (**A**). (**C**) Presents the marker genes corresponding to the spatial domains in (**B**), displayed on spatial coordinates. (**D**) Illustrates the spatial domain identified by computational methods on the 10x Visium human embryo dataset, displayed on spatial coordinates. Each method’s spatial domain is color-coded for clarity, with rough correspondence. (**E**) Provides a separate view of the focused spatial domains and an outer layered structure identified by stMDA, presented on spatial coordinates. The spatial domains are colored as in (**D**). (**F**) Highlights the marker genes corresponding to the spatial domains in (**E**), showcased on spatial coordinates. (**G**) Features a heatmap displaying the marker genes that distinguish the outer layered structure in (**E**). The marker genes are arranged from the outer layer to the inner layer.

For the mouse embryo slice, a previous study meticulously segmented it into 12 distinct domains and provided manual annotations for each. These annotations served as our ground truth for evaluating the spatial domains identified by stMDA and other competing methods on this slice (Fig. 4A and Supplementary Figure 6). Impressively, stMDA’s spatial domains exhibited superior alignment with the manual annotations. This alignment was further reinforced by the concordance with the spatial expression of corresponding marker genes (Fig. 4B and C). Specifically, when considering each domain individually, stMDA and GraphST were the only methods that accurately identified the liver section, characterized by the high expression of the gene *Afp* [27] (Fig. 4A–C). Additionally, stMDA uniquely identified the heart and head mesenchyme, characterized by marker genes *Myl7* and *Crym*, respectively [44]. The precise segmentation achieved by stMDA on the mouse embryo slice attests to its supremacy when handling high-resolution SRT datasets and its consistent performance across platforms.

We further validated the capabilities of stMDA by applying it to a human mouse brain slice profiled using 10x Visium technology. While various methods identified spatial domains with rough similarities, stMDA excelled in capturing finer details, particularly in separating the outer layered structure of the embryo (Fig. 4D and E and Supplementary Figure 7). This separation was defined by marker genes *SCUBE2*, *FABP7*, *COL2A1*, *TAGLN* and *DLK1*, mapping from the outer layer to the inner [45] (Fig. 4G). Notably, stMDA accurately identified the liver and heart regions in the 10x Visium human embryo slice, marked by high expressions of *AFP* and *MYL7*, respectively, consistent with the findings from the Stereo-seq mouse embryo slice (Fig. 4F).

In conclusion, our analysis highlights the robust ability of stMDA to identify spatial domains and reveal corresponding expression patterns, transcending both platforms and species. stMDA proves to be an invaluable tool for dissecting complex anatomical structures across diverse biological contexts.

### stMDA precisely identifies anatomical structures on high-resolution datasets

**Figure 5:**
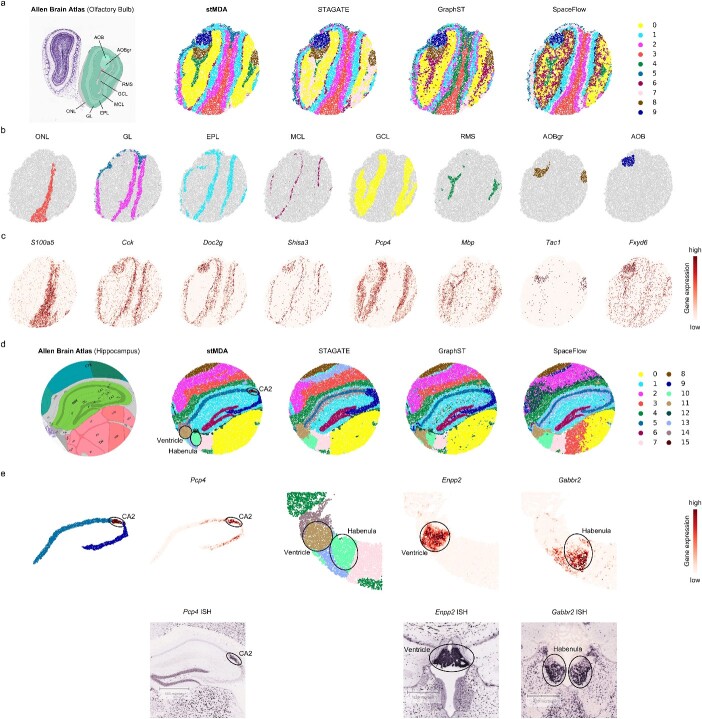
Spatial domain identification on large-scale mouse olfactory bulb and hippocampus datasets profiled by slide-seqV2. (**A**) Displays the anatomical structure from ABA and the spatial domains identified by computational methods on the Slide-seqV2 mouse olfactory bulb dataset, presented on spatial coordinates. Each method’s spatial domain is color-coded for clarity, with rough correspondence. (**B**) Offers a separate view of the focused spatial domains identified by stMDA, as presented on spatial coordinates. The spatial domains are annotated according to the ABA anatomical structure and colored as in (**A**). (**C**) Highlights the marker genes corresponding to the spatial domains in (**B**), showcased on spatial coordinates. (**D**) Depicts the anatomical structure from ABA and the spatial domains identified by computational methods on the Slide-seqV2 mouse hippocampus dataset, exhibited on spatial coordinates. Each method’s spatial domain is color-coded for clarity, with rough correspondence. The focused spatial domains identified by stMDA are highlighted by black circles. (**E**) Provides a separate view, the corresponding marker genes and the corresponding ISH of the focused spatial domains, shown on spatial coordinates. The spatial domains are colored as in (**D**), with the focused spatial domains highlighted by black circles.

We conducted a comprehensive evaluation of stMDA to assess its scalability and robustness using two large-scale, high-resolution Slide-seqV2 datasets—the olfactory bulb and hippocampus [7] (Fig. 5). These sections were chosen due to their well-annotated anatomical structures with clearly defined boundaries from the Allen Brain Atlas (ABA), making them ideal for evaluating the performance of spatial domain identification. It is important to note that Slide-seqV2 datasets present unique challenges, including high resolution, large scale, high dropout rates and elevated noise levels, all of which pose significant computational hurdles.

To begin, we applied stMDA along with other methods (STAGATE, GraphST and SpaceFlow [46]) to identify spatial domains within the Slide-seqV2 mouse olfactory bulb dataset. stMDA effectively identified 10 spatial domains that exhibited better alignment with ABA anatomical structures, featuring fewer noise points and clearer boundaries compared to alternative methods (Fig. 5A and Supplementary Figure 8). These domains were further annotated based on the ABA reference, distinguishing structures from the outer to inner layers: domain 3 as the olfactory nerve layer (ONL), domain 2 and 5 as the glomerular layer (GL), domain 1 as the external plexiform layer (EPL), domain 6 as the mitral cell layer (MCL), domain 0 as the granule cell layer (GCL), domain 4 as the rostral migratory stream (RMS), domain 8 as the granular layer of the accessory olfactory bulb (AOBgr) and domain 9 as the accessory olfactory bulb (AOB). These spatial domains were validated by matching spatial expression patterns with corresponding marker genes such as *S100a5* in ONL [47], *Cck* in GL [48], *Doc2g* in EPL [48], *Shisa3* in MCL [48], *Pcp4* in GCL [49], *Mbp* in RMS [49], *Tac1* in AOBgr [7] and *Fxyd6* in AOB [7] (Fig. 5B and C).

We then extended our analysis to another Slide-seqV2 mouse hippocampus dataset, utilizing stMDA and other methods (Fig. 5D and E). Given the intricacy of the anatomical structures in the hippocampus compared to the olfactory bulb, we identified 16 domains to achieve finer-grained structure distinctions. stMDA’s identification of spatial domains excelled, better delineating anatomical structures in accordance with the ABA reference and displaying enhanced spatial coherence (Fig. 5D and Supplementary Figure 9). Notably, stMDA successfully segmented fine-grained structures, such as cornu ammonis 2 (CA2), Ventricle and Habenula, which were challenging for other methods to capture. The accuracy of stMDA’s identification was further supported by the spatial expression patterns of marker genes and the independent ISH (*in situ* hybridization) image. For instance, the marker gene *Pcp4* exhibited high expression in the CA2 domain, while *Enpp2* and *Gabbr2* were prominently expressed in the Ventricle and Habenula domains, respectively [4] (Fig. 5E).

These collective findings underscore the scalability of stMDA when handling large-scale datasets and its superiority in segmenting anatomical structures, even at a finer-grained level. stMDA’s ability to distinguish intricate structures sets it apart as a powerful tool for spatial analysis across a range of biological contexts.

## Discussion

SRT technologies have significantly advanced our understanding of gene expression by providing important spatial context. However, challenges such as dropouts and noise levels in SRT datasets necessitate the development of computational methods that can effectively integrate gene expression with other modalities, such as histological images and spatial locations, while accounting for technical biases. In response to these challenges, we propose a novel method called stMDA (multi-modal unsupervised domain adaptation), which aims to integrate gene expression and multiple modalities to uncover the spatial functional landscape. stMDA first utilizes multiple neural network architectures to learn representations from spatial multi-modal data and then align the spatial distribution of these modality-specific representations to uncover the spatial functional landscape. Our results demonstrate that stMDA surpasses existing methods in performance and robustness regarding spatial domain identification across various platforms and species. Additionally, stMDA excels in identifying spatially variable genes with high prognostic significance after denoising PDAC cancer slice. These findings underline the promising potential of stMDA as a powerful tool for advancing spatial transcriptomics analysis.

The superior performance of stMDA can be attributed to its distinctive characteristics, i.e. the multi-modal unsupervised domain adaptation and deep spatial distribution alignment scheme. First and foremost, the novelty of the multi-modal unsupervised domain adaptation lies in its utilization of multiple neural network architectures to harness the full potential of their architectural strengths. VAE encoders and decoders specialize in capturing gene expression differences, while GCN encoders excel at incorporating spatial information using an adjacency matrix, and CNN extracts representations from histological images. Moreover, the deep spatial distribution alignment scheme plays a vital role in integrating these neural networks, effectively mitigating the risk of overfitting. Overfitting of the GCN encoders can result in spatially oversmoothed latent embeddings, while overfitting of the VAEs can produce scattered points within the identified spatial domains. Additionally, the distributed alignment is a flexible weak coupling that integrates multi-modal data from the perspective of clustering assignments. In this way, the deep spatial distribution alignment scheme balances these challenges, yielding spatial domains characterized by both spatial continuity and expression characteristics. This stands in contrast to previously proposed computational methods, which are predominantly built on single-model architectures and fail to leverage multiple models for distinct input types and their respective data characteristics. This fundamental departure from single-model approaches addresses a primary limitation in existing methods, thereby contributing to the superior performance of stMDA in spatial transcriptomics analysis.

The architecture of stMDA is both flexible and scalable, enabling the integration of a wide array of biological datasets. In particular, stMDA can simultaneously model expression data, spatial information and histological images, unlocking the potential to identify more biologically informative spatial domains from spatial multi-modal data. Moreover, stMDA’s multi-modal unsupervised domain adaptation model can be seamlessly adapted to handle multi-modal data generated by emerging technologies such as SHARE-seq [50] and CITE-seq [51], thus effectively alleviating the sparse and noisy problem of multi-modal data. In this context, each modality within the multi-modal datasets can be effectively characterized using respective specialized neural network architectures.

**Key Points**

- stMDA is a novel multi-modal unsupervised domain adaptation framework for SRT datasets that precisely reveals the spatial functional landscape and effectively alleviates data sparsity and noisy problem, relying on multiple neural network architectures with cross-modal alignment to faithfully integrate gene expression, histological images and spatial locations.
- stMDA presents an innovative ‘deep spatial distribution alignment’ strategy to significantly reduce the differences of global and spatially local distributions across multi-modal representations, which leads to consistent and reliable clustering assignments.
- stMDA is a powerful tool to provides novel insights in SRT studies and can be scaled for diverse SRT platforms.

## Supplementary Material

stMDA_SI_bbae257

## Data Availability

The human dorsolateral prefrontal cortex (DLPFC) datasets are available in the spatialLIBD package (http://spatial.libd.org/spatialLIBD). The Slide-seqV2 olfactory bulb dataset is available at https://singlecell.broadinstitute.org/single_cell/study/SCP815. The Stereo-seq olfactory bulb dataset is available at https://github.com/JinmiaoChenLab/SEDR_analyses. Python source code of stMDA, under the open-source BSD 3-Clause license, is available at https://github.com/zccqq/stMDA.

## Author Biographies

**Lequn Wang** is pursuing his PhD degree in the Center for Excellence in Molecular Cell Science. His research interests include bioinformatics, machine learning, single-cell multi-omics and spatial transcriptomics.

**Yaofeng Hu** is a master student in Key Laboratory of Systems Health Science of Zhejiang Province, Hangzhou Institute for Advanced Study, University of Chinese Academy of Sciences, Chinese Academy of Sciences, Hangzhou 310024, China. Her current research interests include single-cell transcriptomics and spatial transcriptomics.

**Kai Xiao** is a master student in State Key Laboratory of Cell Biology, Shanghai Institute of Biochemistry and Cell Biology, Center for Excellence in Molecular Cell Science, Chinese Academy of Sciences, Shanghai 200031, China. His current research interests include single-cell transcriptomics and spatial transcriptomics.

**Chuanchao Zhang** received the PHD from Wuhan University, Wuhan, China, in 2017. He is currently an assistant research fellow in Key Laboratory of Systems Health Science of Zhejiang Province, Hangzhou Institute for Advanced Study, University of Chinese Academy of Sciences, Chinese Academy of Sciences, Hangzhou 310024, China. His current research interests include machine learning, deep learning, single-cell transcriptomics and spatial transcriptomics.

**Qianqian Shi** received the PHD from Shanghai Institute of Biological Sciences, University of Chinese Academy of Sciences, Chinese Academy of Sciences, China, in 2017. She is currently an associate professor at College of Informatics, Huazhong Agricultural University, Wuhan, China. Her current research interests include machine learning, deep learning, network biology, computational biology, single-cell transcriptomics and spatial transcriptomics.

**Luonan Chen** received the BS degree from the Huazhong University of Science and Technology, Wuhan, China, in 1984, and the MS and PhD degrees from Tohoku University, Sendai, Japan, in 1988 and 1991, respectively. Since 1997, he has been an Associate Professor at Osaka Sangyo University, Osaka, Japan, where he became a Full Professor. Since 2010, he has been a Professor and the Executive Director with the Key Laboratory of Systems Biology, Shanghai Institute of Biochemistry and Cell Biology, Chinese Academy of Sciences, Shanghai, China. In recent years, he has published over 300 journal papers and two monographs in the area of computational systems biology and nonlinear dynamics. His current research interests include systems biology, bioinformatics and non-linear dynamics.
