# Supplementary material for: Multi-modal domain adaptation for revealing spatial functional landscape from spatially resolved transcriptomics
Source: Brief Bioinform. 2024 May 31;25(4):bbae257. doi: 10.1093/bib/bbae257 (PMC11141295; doi:10.1093/bib/bbae257)
Supplement: stMDA_SI_bbae257 [file stmda_si_bbae257.pdf]

# Multi-modal domain adaptation for revealing spatial functional landscape from spatially resolved transcriptomics

Lequn Wang[1,2], Yaofeng Hu[5], Kai Xiao[1,2], Chuanchao Zhang[5*], Qianqian Shi[3,4*], Luonan Chen[1,2,5*]

[1]Key Laboratory of Systems Biology, Shanghai Institute of Biochemistry and Cell Biology, Center for Excellence in Molecular Cell Science, Chinese Academy of Sciences, Shanghai 200031, China

[2]University of Chinese Academy of Sciences, Beijing 100049, China

[3]Hubei Engineering Technology Research Center of Agricultural Big Data, Huazhong Agricultural University, Wuhan 430070, China

[4]Hubei Key Laboratory of Agricultural Bioinformatics, College of Informatics, Huazhong Agricultural University, Wuhan 430070, China

[5]Key Laboratory of Systems Health Science of Zhejiang Province, School of Life Science, Hangzhou Institute for Advanced Study, University of Chinese Academy of Sciences, Hangzhou 310024, China

* To whom correspondence should be addressed. Chuanchao Zhang, Email: chuanchaozhang@ucas.ac.cn; Qianqian Shi. Email: qqshi@mail.hzau.edu.cn; Luonan Chen Email: lnchen@sibs.ac.cn

# Section S1. Supplementary Notes:

### 1.1 Comparison of other spatial domain identification methods

We adopt the same preprocessing procedure as stMDA for other methods. We implement these methods according to their tutorial websites or code scripts posed on GitHub repository. We implement each method using the default parameters or parameters specified in their tutorials or code scripts with the only exception of adjusting the number of clusters or clustering resolution to guarantee that each method identifies the same number of spatial domains for fair comparison.

The reference implementation of tutorial website or code script for each method is listed below:

- SpatialPCA: https://lulushang.org/SpatialPCA_Tutorial/DLPFC.html
- STAGATE: https://stagate.readthedocs.io/en/latest/T1_DLPFC.html
- BayesSpace: http://www.ezstatconsulting.com/BayesSpace/articles/maynard_DLPFC.html
- DeepST: https://github.com/JiangBioLab/DeepST
- GraphST: https://deepst-tutorials.readthedocs.io/en/latest/Tutorial%201_10X%20Visium.html
- SEDR: https://sedr.readthedocs.io/en/latest/Tutorial1_Clustering.html
- SpaGCN: https://github.com/jianhuupenn/SpaGCN/blob/master/tutorial/tutorial.md
- SpaceFlow: https://github.com/hongleir/SpaceFlow/blob/master/tutorials/seqfish_mouse_embryogenesis.ipynb

We utilize the newest version (until October 2023) of each method to accommodate the new features and improvements. The version of each method is listed below: SpatialPCA v1.3.0; STAGATE v1.0.1; BayesSpace v1.5.1; DeepST code scripts on GitHub; GraphST v1.1.1; SEDR v1.0.0; SpaGCN v1.2.7; SpaceFlow v1.0.3.

### 1.2 Hyperparameter settings

stMDA encompasses several hyperparameters, such as the number of layers in the encoder and the decoder, the number of neurons in each layer, learning rate and the number of training epochs, like other deep learning models. But stMDA is not sensitive to the hyperparameter settings, and we only utilize one set of hyperparameters (default settings) throughout all experiments.

The number of layers in the encoder and the decoder is set to 2 in default. The encoder and the decoder of two layers is sufficient to capture the complex data structure in the spatial transcriptomics and meanwhile maintains the model simplicity. The number of neurons in the hidden layer is set to 128 and the number of neurons in the latent layer is set to 10, which exhibits superior performance throughout all experiments. When it comes to training hyperparameters, we set learning rate to 1e-3 and the number of epochs to 1000, which shows stable convergence throughout all experiments. For large scale datasets, considering mini-batch strategy, the number of input samples per batch is recommended to larger values until GPU is out of memory.

# Section S2. Supplementary Figures and Tables

**Supplementary Figure 1.** The spatial domain identification on the DLPFC dataset to investigate the influence of hyper-parameter λ. Boxplots of the performance of SpaCAE on 12 slices with λ varies from 0 to 2. SpaCAE’ s performance demonstrates a trend of initial improvement followed by a decline, achieving the best performance at λ=1.0.

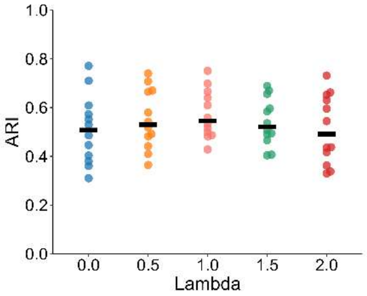

**Supplementary Figure 2.** The manual annotation of 10x Visium DLPFC slice 151507, 151510, 151674 and 161675 on the spatial coordinates. Annotations include L1-L6 (Layer 1-Layer 6) and WM (white matter). The spatial domains identified by stMDA and other competing methods on 10x Visium DLPFC slice 151507, 151510, 151674 and 161675, displayed on the spatial coordinates. Each method's spatial domain is color-coded for clarity, with the ARI value shown above.

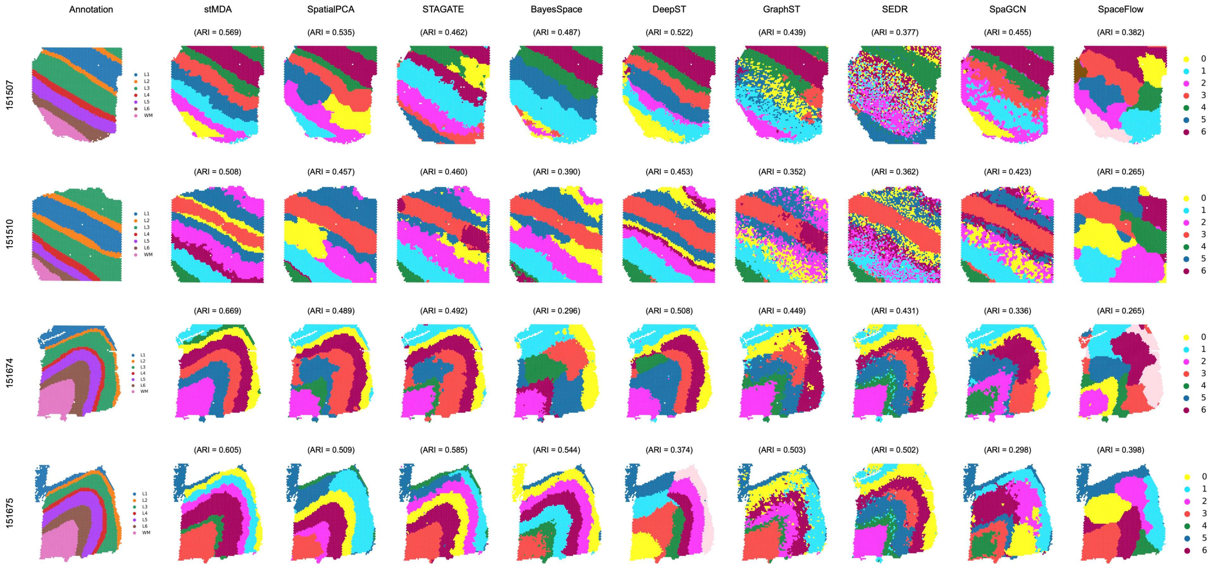

**Supplementary Figure 3.** The spatial domains identified by stMDA and other competing methods on 10x Visium PDAC dataset, displayed on the spatial coordinates. Each method's spatial domain is color-coded for clarity, with the SC and DB value shown above. SC, silhouette score; DB, Davies-Bouldin score.

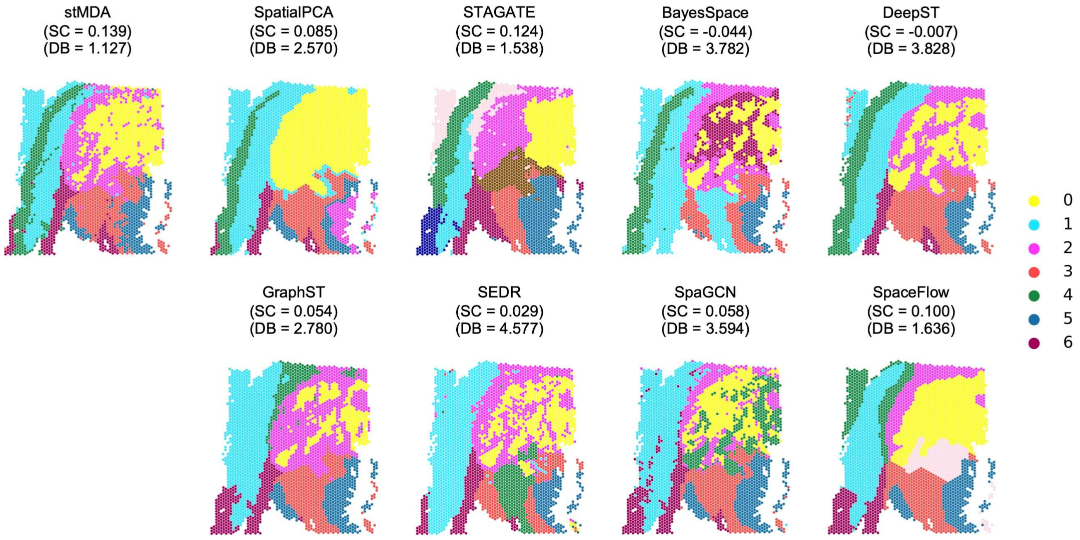

**Supplementary Figure 4.** The marker genes of pancreatic ductal adenocarcinoma tumors and In situ staining of marker genes.

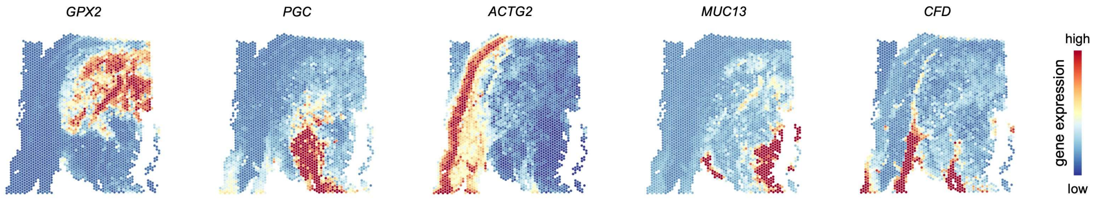

**Supplementary Figure 5.** The boxplot of Moran's I value, measuring the spatial autocorrelation pattern before and after denoising on 10x Visium PDAC dataset. In the boxplot, the center line represents the median, the box limits indicate the upper and lower quartiles, and whiskers represent the 1.5× interquartile range.

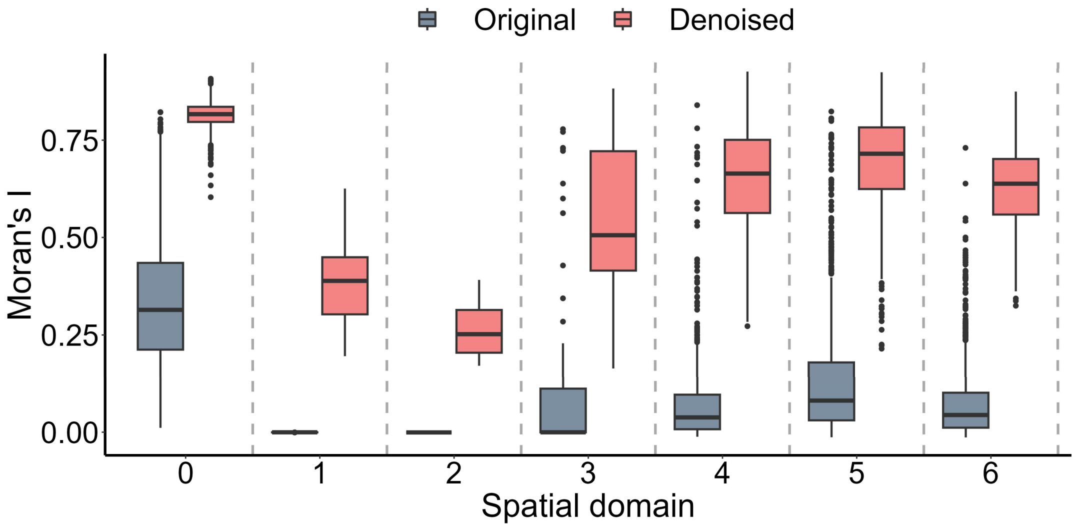

**Supplementary Figure 6.** The manual annotation and the spatial domains identified by stMDA and other competing methods on Stereo-seq mouse embryo slice, displayed on the spatial coordinates. Each method's spatial domain is color-coded for clarity, with the SC and DB value shown above. SC, silhouette score; DB, Davies-Bouldin score.

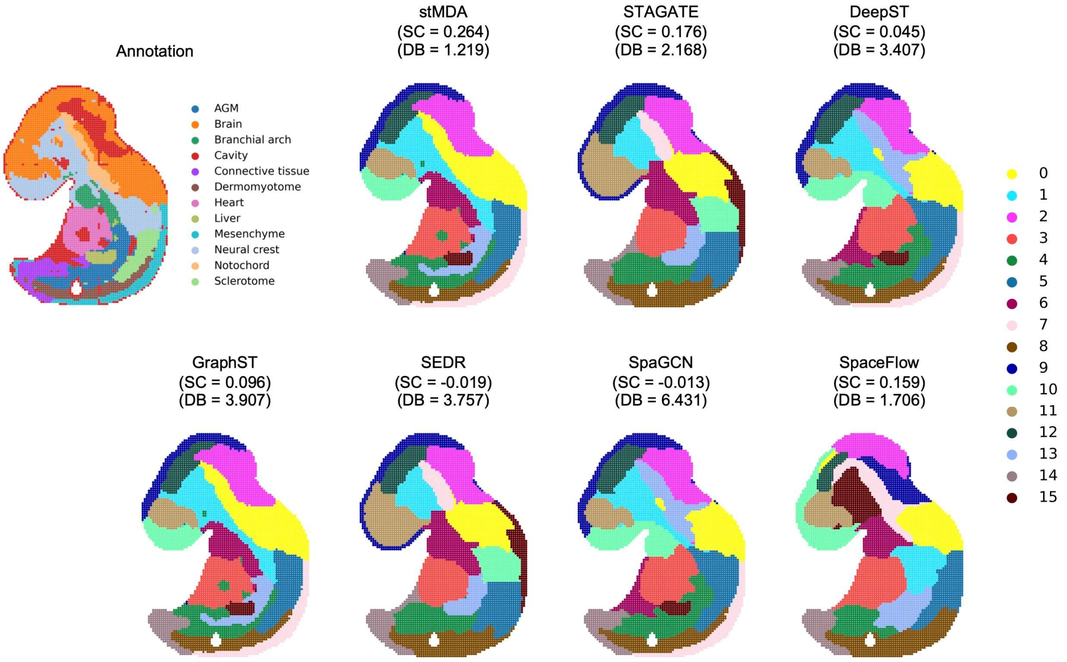

**Supplementary Figure 7.** The spatial domains identified by stMDA and other competing methods on 10x Visium human embryo dataset, displayed on the spatial coordinates. Each method's spatial domain is color-coded for clarity, with the SC and DB value shown above. SC, silhouette score; DB, Davies-Bouldin score.

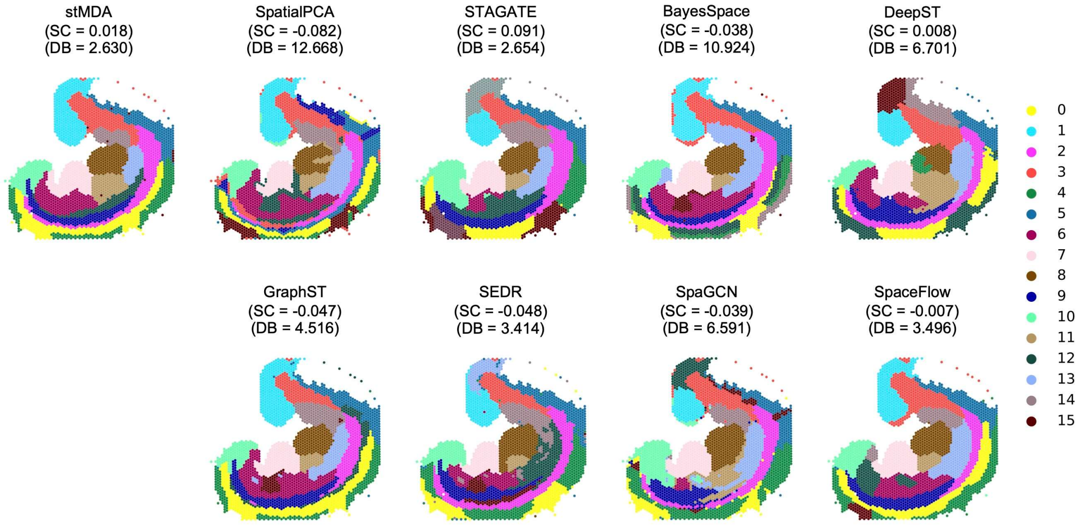

**Supplementary Figure 8.** The anatomical structure from Allen Brain Atlas and the spatial domains identified by computational methods on the Slide-seqV2 mouse olfactory bulb dataset, presented on spatial coordinates. Each method's spatial domain is color-coded for clarity, with the SC and DB value shown above. SC, silhouette score; DB, Davies-Bouldin score.

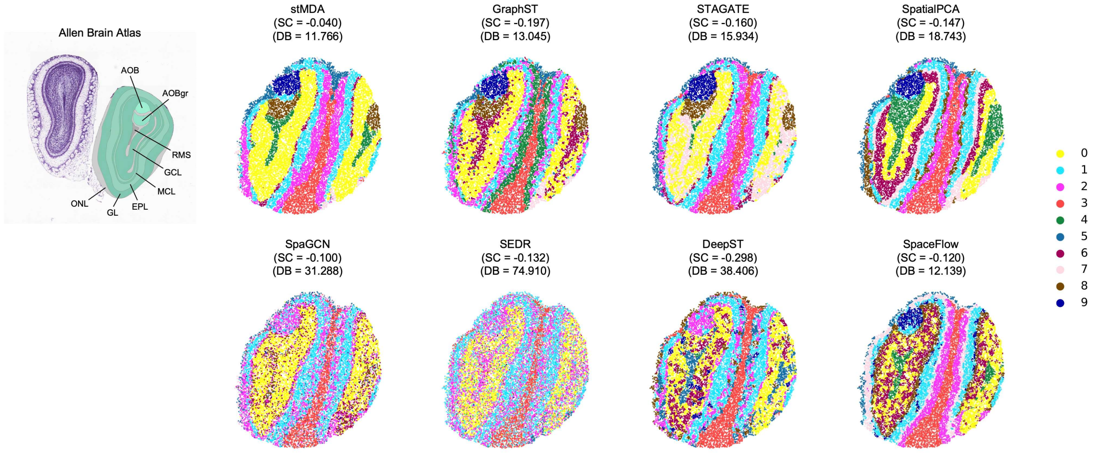

**Supplementary Figure 9.** The anatomical structure from Allen Brain Atlas and the spatial domains identified by computational methods on the Slide-seqV2 mouse hippocampus dataset, presented on spatial coordinates. Each method's spatial domain is color-coded for clarity, with the SC and DB value shown above. SC, silhouette score; DB, Davies-Bouldin score.

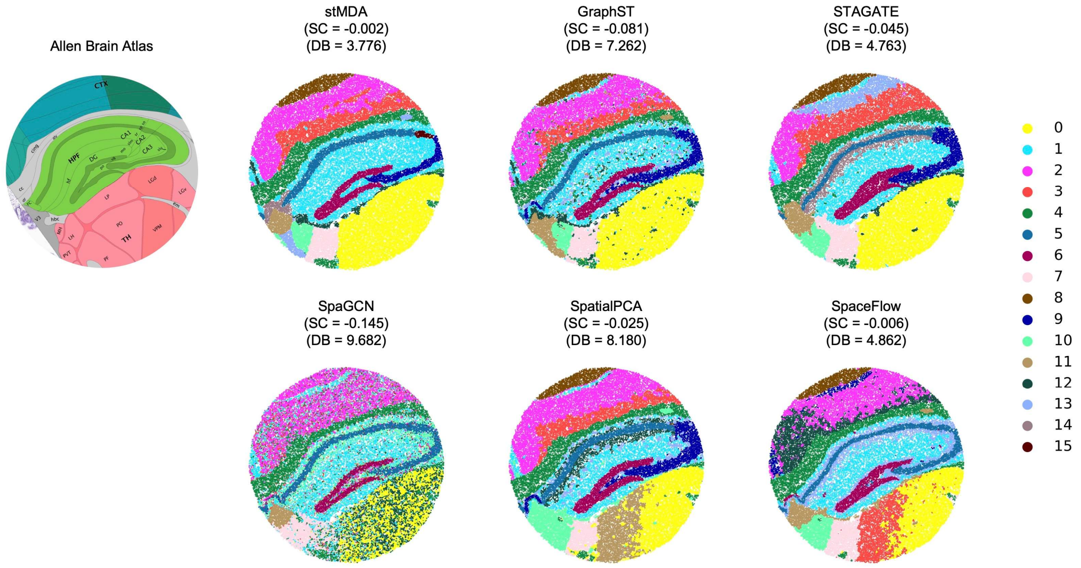

**Supplementary Table 1.** Detailed settings of parameter λ in the SRT data from different platforms.

| Dataset | $\lambda$ |
| --- | --- |
| 10x Visium | 0.5~2 |
| Slide-seqV2 | 1~3 |
| Stereo-seq | 1~3 |
